# Feedback regulation of apical progenitor fate by immature neurons through Wnt7–Celsr3–Fzd3 signalling

**DOI:** 10.1038/ncomms10936

**Published:** 2016-03-04

**Authors:** Wei Wang, Yves Jossin, Guoliang Chai, Wen-Hui Lien, Fadel Tissir, Andre M. Goffinet

**Affiliations:** 1 Université catholique de Louvain, Institute of Neuroscience, 73 Avenue Mounier, Box B1.7316, 1200 Brussels, Belgium; 2 Université catholique de Louvain, de Duve Institute, Avenue Hippocrate 74, Box B1.74.09, 1200 Brussels, Belgium; 3 WELBIO, 6 Avenue Pasteur, 1300 Wavre, Belgium

## Abstract

Sequential generation of neurons and glial cells during development is critical for the wiring and function of the cerebral cortex. This process requires accurate coordination of neural progenitor cell (NPC) fate decisions, by NPC-autonomous mechanisms as well as by negative feedback from neurons. Here, we show that neurogenesis is protracted and gliogenesis decreased in mice with mutations of genes *Celsr3* and *Fzd3*. This phenotype is not due to gene inactivation in progenitors, but rather in immature cortical neurons. Mutant neurons are unable to upregulate expression of Jag1 in response to cortical Wnt7, resulting in blunted activation of Notch signalling in NPC. Thus, Celsr3 and Fzd3 enable immature neurons to respond to Wnt7, upregulate Jag1 and thereby facilitate feedback signals that tune the timing of NPC fate decisions via Notch activation.

## Abstract (web summary)

The switch from neurogenesis to gliogenesis in cortical development is only partially understood. Here the authors show that Wnt-Planar cell polarity signaling in immature cortical neurons activates Notch in neural progenitor cells, thereby tuning the timing of their fate decisions.

## Graphical abstract

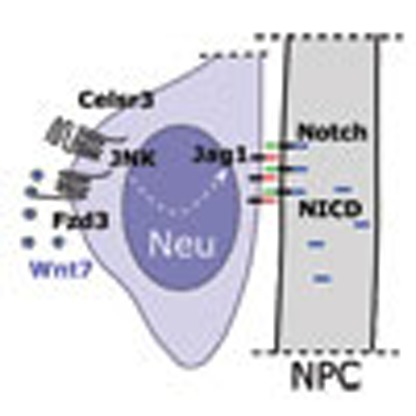

During cortical development, neuroepithelial/apical neural progenitor cells (AP) undergo divisions that are initially symmetrical and increase the size of the progenitor pool. At the onset of neurogenesis, some divisions become asymmetric and generate AP and either neurons or intermediate/basal progenitors (BP) which have limited self-renewal capacity and are committed to an excitatory glutamatergic neuron phenotype^1^,^2^,^3^,^4^,^5^. In the dorsal telencephalic wall, neural progenitor cells (NPC, including AP and BP) sequentially generate deep layer (DL, mostly layers 5 and 6) and upper layer (UL, mostly layers 2, 3 and 4) neurons, followed by glial cells. Despite significant progress, the mechanisms that regulate the switch between production of DL and UL neurons, and the neurogenic to gliogenic switch remain incompletely known^6^. The sequential formation of cortical neurons and glia can be recapitulated *in vitro* in minimal medium^7^,^8^,^9^, suggesting that NPC-autonomous events play a critical role. An internal clock mechanism, however, can hardly provide sufficient plasticity, and additional regulation is provided by the micro-environment, such as signals from meninges, growth factors in cerebrospinal fluid^10^ and feedback from BP and postmitotic neurons to AP (refs 11−15, 12, 13, 14, 15).

The neurogenic to gliogenic switch is regulated by various signalling pathways^13^. Jak/Stat cytokines such as cardiotrophin-1 and CNTF produced by postmitotic neurons promote the production of glia^16^,^17^,^18^, and NPC deficient in both Mek1 and Mek2 fail to switch from neurogenesis to gliogenesis due to attenuation of the cytokine-regulated gliogenic pathway^19^. Neurotrophin-3 (Ntf3) and Fibroblast Growth Factor-9 produced by neurons in response to the transcriptional regulator Sip1 influence NPC fate^12^,^14^. Bmp2 and Bmp4 promote astrocytogenesis from NPC^20^. Unlike the gliogenic switch, how extrinsic factors regulate the sequential formation of cortical neurons is less well understood. Feedback from DL neurons to AP may scale production of UL neurons^21^, and some mechanisms may be common to the neuro- and gliogenic switches: for example, the Ntf3 feedback also regulates the transition from DL to UL neuron formation^12^.

Particularly prominent among the pathways that regulate cortical development is Notch signalling^22^,^23^,^24^,^25^. Notch1–4 receptors on AP cells can be activated by ligands Delta (Dll1,3,4) or Jagged (Jag1, 2) on adjacent cells such as other AP located in ventricular zones or BP located in the subventricular zone^15^. Those interactions play important roles in maintaining the AP population and inhibiting premature generation of neurons, which is not solely explained by regulation of differentiation timing^25^. At later stages, they have a crucial role in promoting gliogenesis^11^. Of note, the regulation of neurogenesis by the six transmembrane domain protein Gde2 and its substrate RECK (ref. 26) has been attributed to their interactions with Notch, further emphasizing its master role.

Planar cell polarity (PCP) in epithelial sheets is regulated by various genes, among which the so called ‘core PCP' genes include the seven pass transmembrane domain receptors Fzd3 and 6, the atypical seven pass cadherins Celsr1–3, the tetraspannins Vangl1 and 2 and the adaptors Dishevelled (Dvl)1–3 and Prickle^27^,^28^. Celsr3 and Fzd3, in particular, are required for axon guidance, neuronal migration and ependymal cilia development^29^. Here we show that Celsr3 and Fzd3-deficient cerebral cortex is characterized by increased neuronal and decreased glial density, indicating possible defective timing of neurogenesis and gliogenesis in AP. Importantly, this is caused by absence of Celsr3 or Fzd3 in immature neurons, not in AP. Celsr3 and Fzd3-deficient neurons express less Jag1 than control ones, and fail to activate Notch properly in AP, resulting in increased neurogenesis and decreased gliogenesis, which is rescued on overexpression of Jag1. Jag1 messenger RNA (mRNA) is upregulated in cultured cortical neurons on treatment by Wnt7, the most abundant Wnt factor in the embryonic cortex, and this effect is blunted in *Celsr3* and *Fzd3* mutant neurons. Thus, Celsr3 and Fzd3 are required in immature neurons and possibly BP, to upregulate Jag1 in response to Wnt7, and to activate Notch signalling in AP, providing a feedback signal to tune AP cell fate and timing transitions.

## Results

### Neurogenesis is increased in *Celsr3* and *Fzd3* mutant cortex

**Figure 1:**
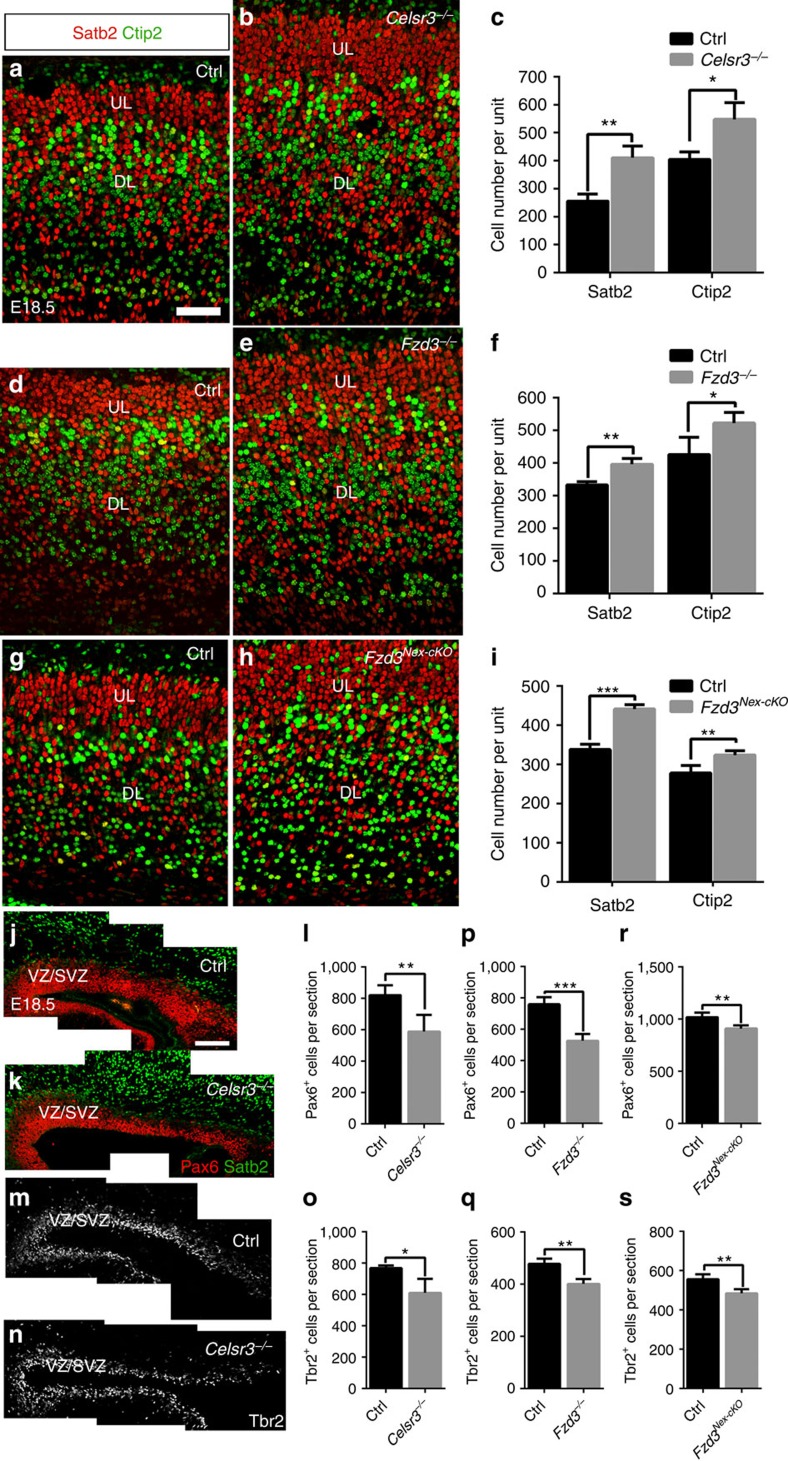
Neurons are increased and NPC decreased in mutant cortex. (**a**–**i**) Both DL neurons (Ctip2) and UL neurons (Satb2) are increased in *Celsr3* (**a**–**c**), *Fzd3* (**d**–**f**) and *Fzd3*^*Nex-cKO*^ (**g**–**i**) mutant cortex. Scale bar, 50 μm. Number of animals: 3 Ctrl and 3 *Celsr3* (**c**); 3 Ctrl and 4 *Fzd3* (**f**); 4 Ctrl and 4 *Fzd3*^*Nex-cKO*^ (**i**). Unpaired *t*-test. Error bars: s.d. (**j**–**s**) The number of apical progenitors (Pax6, **j**–**l**) and to a lower extent BPs (Tbr2, **m**–**o**) is decreased in *Celsr3*, as well as in *Fzd3*^*−/−*^ (**p**,**q**) and *Fzd3*^*Nex-cKO*^ mutant cortex (**r**,**s**). VZ/SVZ, ventricular and subventricular zone. Scale bar, 100 μm. Number of animals: 4 Ctrl and 4 *Celsr3* (**l**); 4 Ctrl and 3 *Celsr3* (**o**); 4 Ctrl and 4 *Fzd3* (**p**,**q**); 4 Ctrl and 4 *Fzd3*^*Nex-cKO*^ (**r**,**s**). Unpaired *t*-test. Error bars: s.d.

To assess the formation of cortical neurons, we examined brains at a late embryonic stage (E18.5) using layer-specific markers Tbr1 (layer 6), Ctip2 (layers 5–6) and Satb2 and Cux1 (layers 2–4). Compared with control samples, the number of Ctip2^+^ and Satb2^+^ cells was increased in *Celsr3* (Fig. 1a–c) and *Fzd3* (Fig. 1d–f) mutant cortices, with increased thickness of the cortical plate (CP), whereas tangential expansion did not affect the cortical ribbon and was restricted to germinal layers (Supplementary Fig. 1). This was confirmed in Tbr1 and Cux1-stained preparations (Supplementary Figs 2 and 3). Of note, despite increased cortical neuron numbers, the border between Satb2 and Ctip2-positive layers was sharply defined in mutant cortex, indicating that neuronal migration and lamination were unaffected. Previous mRNA *in situ* hybridization data showed that *Celsr3* is specifically expressed in postmitotic neurons, with some expression in BP, but not in AP, whereas *Fzd3* is widely expressed in both NPCs and neurons^30^. The similar cortical alterations in *Celsr3* and *Fzd3* mutants are therefore probably due to inactivation of Fzd3 in immature cortical neurons or BP, rather than in AP. To ascertain this, we produced conditional *Fzd3*^*f/−*^*; Nex-Cre* mutant animals (*Fzd3*^*Nex-cKO*^), in which *Fzd3* is specifically inactivated on expression of *Nex-Cre* in cortical excitatory neurons^31^. To verify Cre activity, we cut tangentially cortical wall samples to separate ventricular zones and subventricular zone on one hand, and cortical plate on the other hand, and carried out PCR with reverse transcription (RT-PCR) with primers in exon 3, which is deleted in our *Fzd3* mutant allele. As shown in Supplementary Fig. 4, comparable RT-PCR signals were obtained from ventricular zones and cortical plate in control tissue, whereas no amplicon was obtained from *Fzd3*^*Nex-cKO*^ cortical plate tissue. In *Fzd3*^*Nex-cKO*^ animals, increased production of DL and UL cortical neurons was observed, like in *Celsr3*^*−/−*^ and *Fzd3*^*−/−*^ mutant embryos (Fig. 1g–i). In contrast to the cortical plate, the ventricular zones and subventricular zone were thinner and tangentially expanded in mutants, as described before^32^,^33^ (Fig. 1j–s, Supplementary Fig. 1). To estimate the number of AP in mutant and control samples, we used the AP marker Pax6. There was a significant decrease of the number of Pax6^+^ cell in every section in *Celsr3*^*−/−*^ (Fig. 1j–l), *Fzd3*^*−/−*^ (Fig. 1p) and *Fzd3*^*Nex−cKO*^ (Fig. 1r) mutant versus control samples. As cortical neurons derive from both AP and BP, we wondered whether the number of BP, which express Celsr3 at lower levels than neurons, was also affected. Using immunohistochemistry (IHC) with the BP marker Tbr2, we found that the number of BP was modestly decreased in *Celsr3*^*−/−*^, *Fzd3*^*−/−*^ and *Fzd3*^*Nex-cKO*^ mutants versus control cortex (Fig. 1m–o,q,s), albeit less significantly than that of Pax6-positive AP. These observations of an excess of both DL and UL cortical neuron numbers with depletion of NPC numbers may indicate defective feedback regulation.

**Figure 2:**
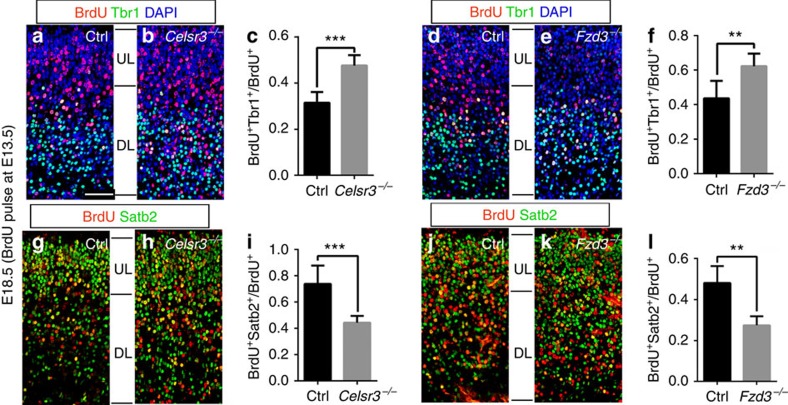
Generation of DL neurons is increased in mutants. (**a**–**f**) Double staining at E18.5 for BrdU (injected at E13.5) and Tbr1 shows that there are less double labelled DL neurons in control (**a**,**c**,**d**,**f**) than in *Celsr3* (**b**,**c**) and *Fzd3* (**e**,**f**) cortical sections. Number of samples: 6 Ctrl and 6 *Celsr3* sections from three animals (**c**); 7 Ctrl and 8 *Fzd3* sections from three animals (**f**). Unpaired *t*-test. Error bars: s.d. **(g**–**l**) Double staining at E18.5 for BrdU (injected at E13.5) and Satb2 shows that there are more double labelled UL neurons in control (**g**,**i**,**j**,**l**) than in *Celsr3* (**h**,**i**) and *Fzd3* (**k**,**l**) cortical sections. Areas illustrated correspond to the frame shown in Supplementary Fig. 1a. Scale bar, 50 μm. Number of samples: 8 Ctrl and 7 *Celsr3* sections from three animals (**i**); 5 Ctrl and 5 *Fzd3* sections from two animals (**l**). Unpaired *t*-test. Error bars: s.d.

We looked at earlier time points to determine when the phenotype of increased neurogenesis was first observed, and found that the expression of Ctip2 was comparable in mutant and control samples at E14.5, and that its relative increase in mutants was first noticed at E15.5 and maintained at later stages (Supplementary Fig. 5), indicating that the generation of DL was increased in mutants. To define further the increased production of DL neurons in mutants, 5-bromodeoxyuridine (BrdU) was injected at E13.5, and animals were killed at E18.5. Tbr1 or Satb2 were used to co-stain with anti-BrdU. We counted only cells with strong nuclear BrdU labelling, considered to be born at E13.5, from the first cell division after tracer injection. The proportions of BrdU^+^ cells expressing Tbr1 or Satb2 over the total number of BrdU^+^ cells were determined. The results showed that in *Celsr3* and *Fzd3* mutant cortex, the ratio of BrdU^+^ Tbr1^+^ to all BrdU^+^ cells was increased (Fig. 2a–f, Supplementary Fig. 6), whereas the proportion of BrdU^+^ cells that expressed the UL marker Satb2 was decreased in both *Celsr3* and *Fzd3* mutant samples (Fig. 2g–l). The observation that generation of DL neurons is increased and that of UL neurons decreased indicates that the former is prolonged or amplified in mutants.

**Figure 3:**
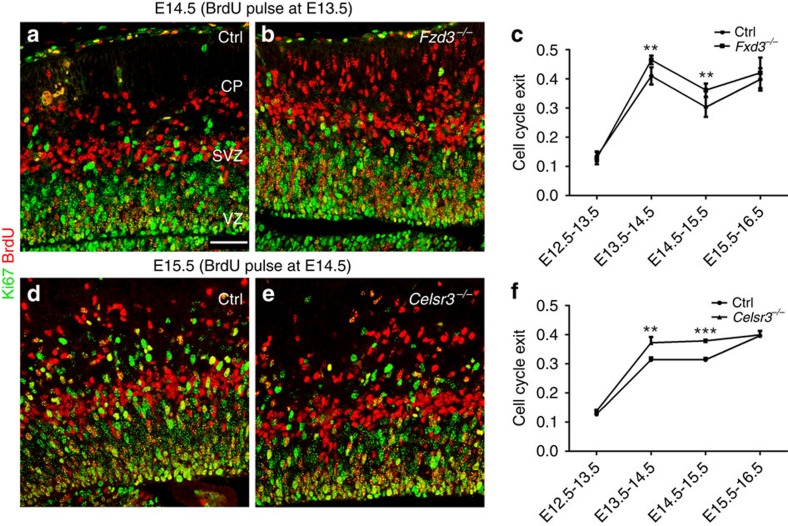
Cell cycle exit is increased in *Celsr3* and *Fzd3* mutants. (**a**–**f**) The rate of NPC cell cycle exit (ratio of BrdU^+^;Ki67^−^/BrdU^+^cells) is increased from E13.5 to E14.5 in *Fzd3* (**a**–**c**) and *Celsr3* (**d**–**f**) mutant cortex. SVZ, subventricular zone; VZ, ventricular zone. Scale bar, 50 μm. Sample numbers: three embryos of each genotype at each stage (**c**,**f**). Unpaired *t*-test. Error bars: s.d.

Several mechanisms could account for the observed increase of neuron number. It has been proposed that thalamocortical afferents influence neurogenesis in some context^34^. To test a potential role of thalamic fibres, we examined *Celsr3*^*Dlx-cKO*^ mice in which these fibres do not develop^35^ and confirmed that the numbers of DL and UL neurons were similar to controls, arguing against a role of thalamic fibres in the phenotype. Another possibility could be differential cell death in mutant and control brains. To assess whether apoptosis was involved in the cortex of *Celsr3* and *Fzd3* mutants, we used IHC with activated Caspase-3 (aCas3), and found very few aCas3-positive cells, with no difference between control, *Celsr3* and *Fzd3* mutant samples at E14.5 and E16.5 (not shown). A third way to increase neuron numbers could be premature NPC cycle exit and/or changes in cell cycle length. To estimate NPC cell cycle exit, we injected BrdU at different stages, from E12.5 to E15.5, and killed animals 24 h later. BrdU and Ki67 double IHC was used to calculate cell cycle exit rates^36^. We found no differences between control and mutant samples injected at E12.5 and examined at E13.5. By contrast, in samples injected at E13.5 and studied at 14.5 (Fig. 3a–c,f), and in those injected at E14.5 and processed at E15.5 (Fig. 3c–f), the cell cycle exit rate was significantly increased in *Celsr3* and *Fzd3* mutants as compared with controls. To compare cell cycle length, we injected 5-bromo-2′-deoxyuridine (BrdU) followed by 5-ethynyl-2′-deoxyuridine (EdU), collected samples 30min later, and estimated S-phase and cell cycle length as described^37^. No differences were found between control, *Celsr3* and *Fzd3* mutant cortex (Supplementary Fig. 7).

### Gliogenesis is decreased in *Celsr3* and *Fzd3* mutant cortex

**Figure 4:**
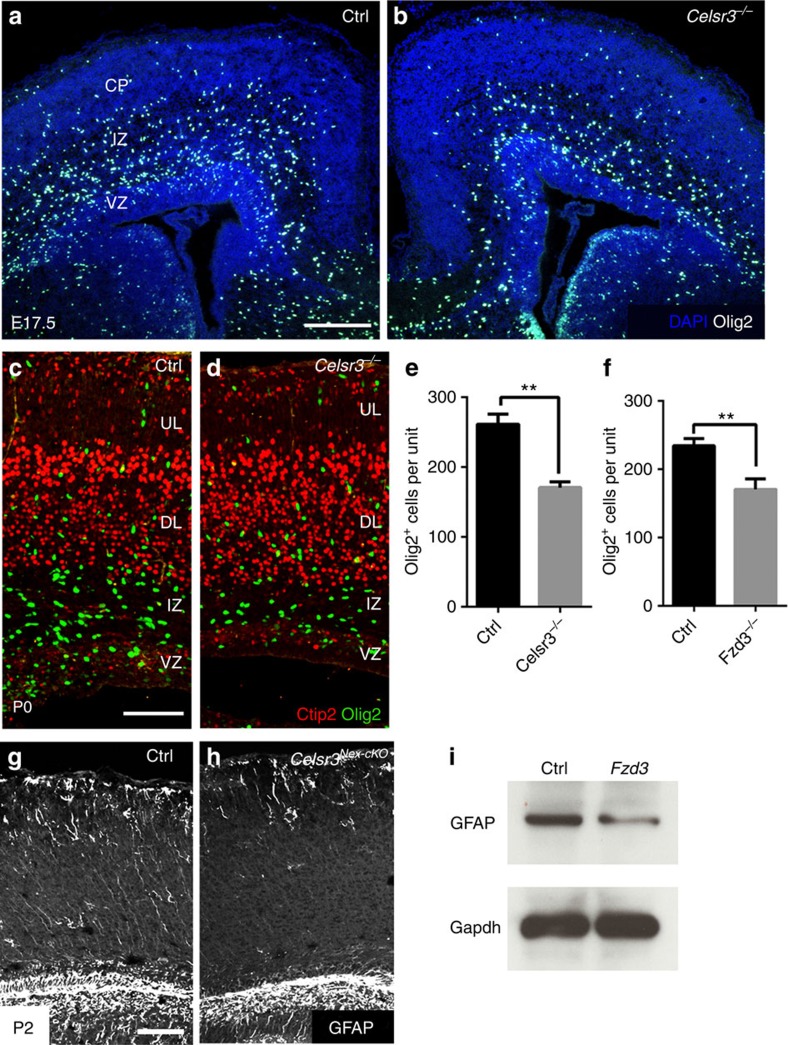
Glial precursors and glial cells are decreased in *Celsr3* and *Fzd3* mutants. (**a**,**b**) Low power view of Control (**a**) and *Celsr3* mutant (**b**) cortex at E17.5 showing decreased number of Olig2^+^ cells (white) in mutant versus control tissue. IZ, intermediate zone; VZ, ventricular zone. Scale bar, 100 μm. (**c**–**f**) At P0, the number of Olig2^+^ cells (green) is decreased in *Celsr3* and *Fzd3* mutants (**P<0.01, unpaired *t*-test). Scale bar, 100 μm. Number of animals: 5 Ctrl and 5 *Celsr3* (**e**); 3 Ctlr and 3 *Fzd3* (**f**). Unpaired *t*-test. Error bars: s.d. (**g**,**h**) At P2, GFAP immunoreactivity is decreased in cortex of *Celsr3*^*Nex-cKO*^ mutants compared to control. Scale bar=100 μm. (**i**) At P0, GFAP protein concentration is decreased in *Fzd3* mutant cortex (Gapdh as internal control).

**Figure 5:**
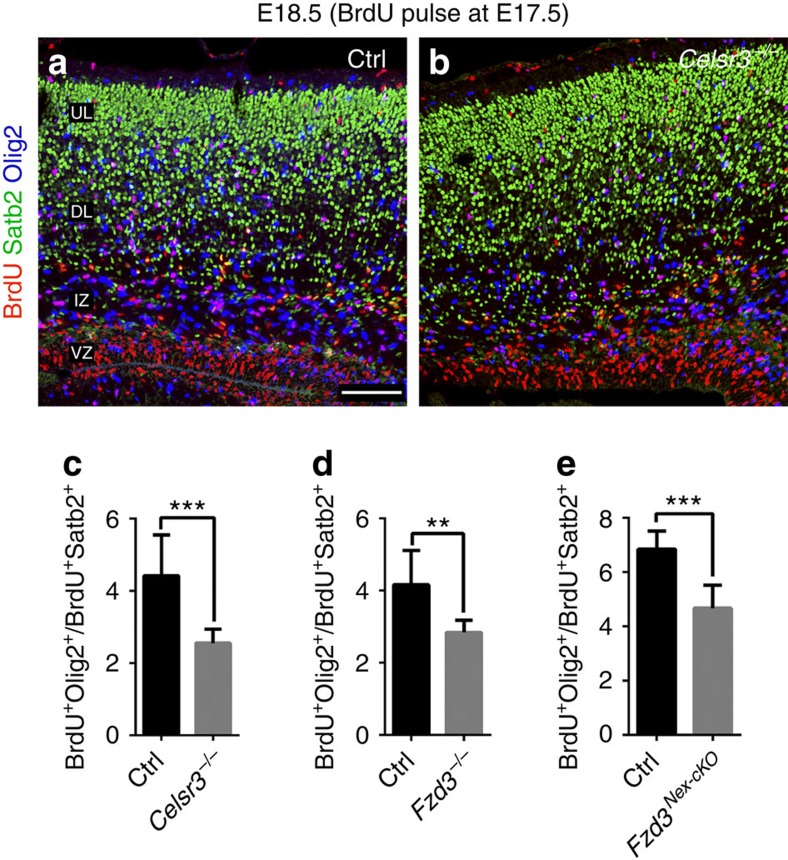
The gliogenic switch is delayed in *Celsr3*, *Fzd3* and *Fzd3*^*Nex-cKO*^ cortex. (**a**–**e**) Using triple IHC for Satb2 (neurons, green), Olig2 (glia precursors, blue) and BrdU (24 h labelling, red) the relative numbers of BrdU^+^ Olig2^+^ to BrdU^+^ Satb2^+^ cells are decreased in all three mutants (**c**–**e**). IZ, intermediate zone; VZ, ventricular zone. Scale bar, 50 μm. Number of samples: 10 Ctrl and 9 *Celsr3* sections from three animals (**c**); 9 Ctlr and 7 *Fzd3* sections from three animals (**d**); 7 Ctlr and 10 *Fzd3*^*Nex-cKO*^ sections from three animals of each genotype (**e**). Unpaired *t*-test. Error bars: s.d.

In the mouse cortex, at E16.5–E17.5, the generation of neurons declines and that of glial cells begins, the so called ‘gliogenic switch'^13^. We considered the possibility that the observed increase of cortical neurons may coincide with a delayed or decreased generation of glial cells. Cortical astrocytes and oligodendrocytes are derived from Olig2^+^ glial precursor cells^6^,^14^. We compared Olig2 expression in control and mutants and found that Olig2^+^ cells were more abundant in control than in *Celsr3* and *Fzd3* mutants at E17.5 (Fig. 4a,b) and P0 (Fig. 4c–f). This indicates that neurons are generated at the expense of glial cells. To confirm this, we used the glial fibrillary acidic protein (GFAP) immunohistochemistry (IHC) at postnatal stages P0 and P2. The intensity of GFAP staining was strongly decreased in mutant cortices (Fig. 4g,h), as was the concentration of GFAP, estimated using western blotting (Fig. 4i). To assess further the relative production of neurons and glia, we administered BrdU at E17.5, and killed animals 24 h later. Using triple IHC for BrdU, Satb2 to label late-generated neurons, and Olig2 to label glial precursors (Fig. 5a,b), we found that the ratio between glial cells and neurons decreased in *Celsr3*, *Fzd3* and *Fzd3*^*Nex-cKO*^ mutant versus control samples (Fig. 5c–e). These data suggest that the neurogenesis may be prolonged or increased, and gliogenesis delayed or defective in mutant animals.

### Jag1-Notch activity is attenuated in *Celsr3* and *Fzd3* mutants

**Figure 6:**
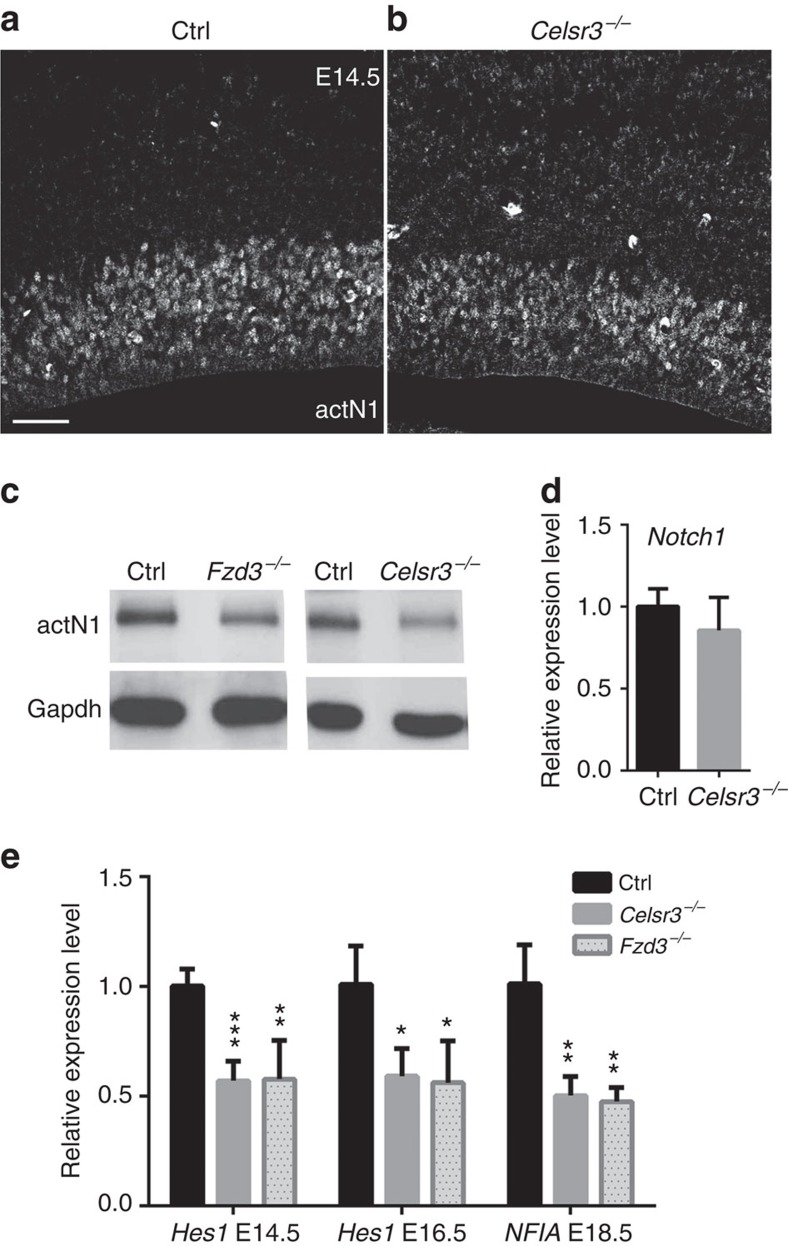
Notch activity is decreased in mutant cortical AP. (**a**,**b**) Using actN1 IHC at E14.5, signal intensity in ventricular zones are lower in mutant (**b**) than control samples (**a**). Scale bar, 50 μm. (**c**) Western blot analysis at E18.5 shows that actN1 immunoreactivity is lower in *Fzd3* and *Celsr3* mutants than in control tissue; Gapdh is internal control. (**d**) *Notch1* mRNA expression is unaffected by *Celsr3* mutation. Number of samples: three animals of each genotype. Unpaired *t*-test. Error bars: s.d. (**e**) Notch targets *Hes1* and *NFIA* are decreased at E14.5 and E16.5, and at E18.5, respectively, in *Celsr3* and *Fzd3* mutants. Number of samples: four embryos of each genotype and stage. Unpaired *t*-test. Error bars: s.d.

To explore mechanisms, we first asked whether the main signalling pathways implicated in the gliogenic switch^13^ could be modified in *Celsr3* and *Fzd3* mutant tissue. We used western blotting with antibodies to phosphorylated Stat3, a readout of Jak-Stat signalling^17^, phosphorylated Erk1 and 2 (ref. 19), and phosphorylated Smads, an index of Bmp signalling^20^. No difference was found between control, *Celsr3* or *Fzd3* mutants (Supplementary Fig. 8), suggesting that those signalling pathways are unaffected by inactivation of Celsr3 or Fzd3 in cortical neurons. We next focused on Notch signalling, a master regulator of neuro- and gliogenesis^22^,^23^. We tested Notch activation at different embryonic stages, from E14.5 to E18.5, by IHC and western blotting with an antibody against the N-terminal epitope of the Notch1 intracellular domain (actN1) generated on cleavage by the gamma secretase complex. IHC at E14.5 and E16.5 disclosed specific actN1 immunoreactivity in AP but no specific signal in postmitotic neurons (Fig. 6a, Supplementary Fig. 9a). The signal was consistently and strongly decreased in *Celsr3* mutant compared with control cortex (Fig. 6b, Supplementary Fig. 9b). Western blot analysis at E18.5 confirmed the downregulation of actN1 signal in *Celsr3* and *Fzd3* mutant versus control samples (Fig. 6c). Of note, *Notch1* mRNA levels, estimated by quantitative RT-PCR (qRT-PCR), remained unchanged, indicating that the modification of actN1 signals did not reflect decreased *Notch1* gene expression (Fig. 6d). In addition, transcripts of the Notch downstream effectors *Hes1*, implicated in neurogenesis^24^, and *NFIA*, implicated in gliogenesis^11^ were downregulated in both *Celsr3* and *Fzd3* mutant embryonic cortex (Fig. 6e), providing another independent evidence that Notch signalling is altered in mutants.

**Figure 7:**
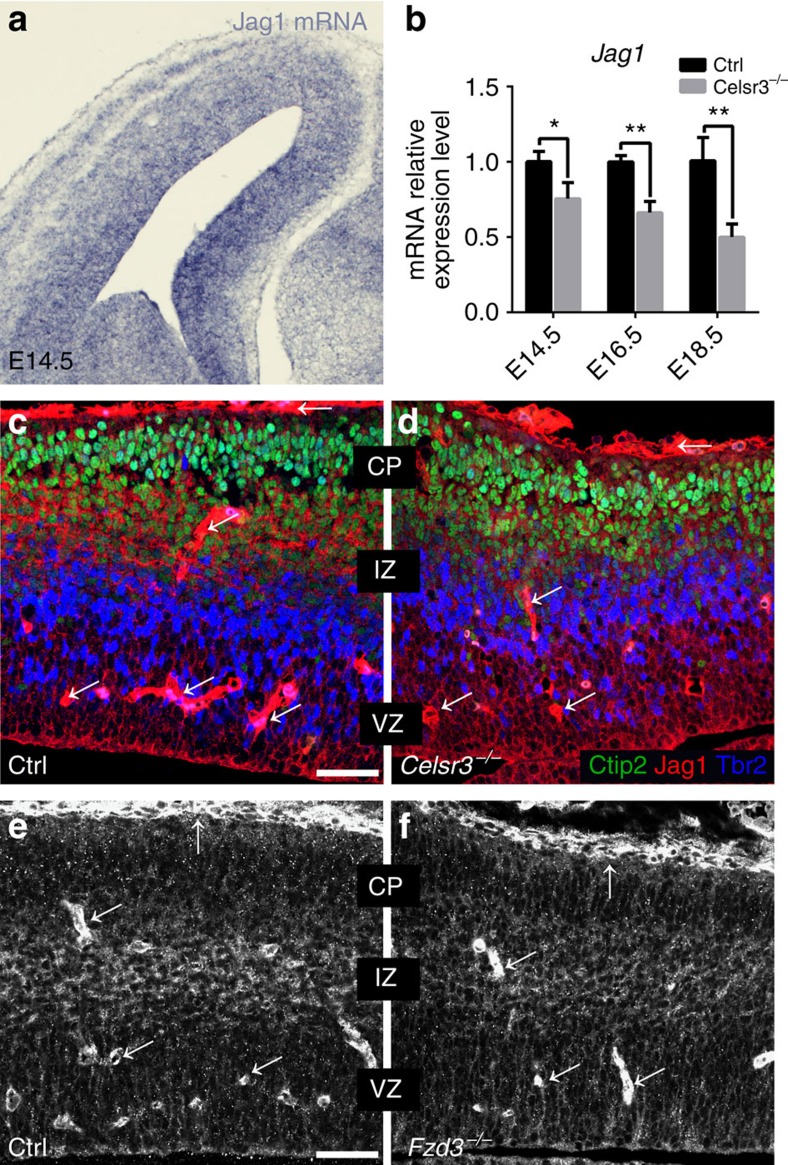
Jag1 is downregulated in mutant cortical neurons. (**a**) *In situ* hybridization at E14.5 shows that *Jag1* mRNA is present in both NPC and early neurons. (**b**) *Jag1* mRNA expression, assessed by qRT-PCR, is decreased in *Celsr3* mutant tissue. (**c**,**d**) Triple IHC at E14.5 with Ctip2, Tbr2 and Jag1 antibodies shows that most of the Jag1 signal is associated with Ctip2, whereas only very few Tbr2^+^ cells are Jag1^+^ (**c**). In *Celsr3*^*−/−*^ mutants, the Jag1 signal decreases in neurons, whereas signal associated with ventricular zone (VZ) and vessels (arrows) is unchanged (**d**). Scale bar, 50 μm. Number of samples: three embryos of each genotype and stage. Unpaired *t*-test. Error bars: s.d. (**e**,**f**) A similar downregulation of Jag1 in neurons is observed in *Fzd3*^*−/−*^ mutant (**f**) compared with control (Ctrl, **e**). IZ, intermediate zone; VZ, ventricular zone. Scale bar, 50 μm.

What mechanisms could account for decreased Notch1 activation in *Celsr3* and *Fzd3* mutants? To assess the role of Notch ligands, we used an RNA-Seq data set (Methods section) comparing transcriptional profiles in control, *Celsr3* and *Fzd3* mutant cortical samples at E16.5. Among Notch ligands, *Dll1* mRNA was strongly expressed in all samples, whereas *Dll3*, *Dll4*, *Jag1* and *Jag2* were moderately represented. *Jag1* was the sole mRNA significantly downregulated in both *Fzd3* (by 26%, *P*=0.03) and *Celsr3* mutant samples (by 27%, *P*=0.02); it was therefore the prime candidate, and selected for further study. *Jag1* mRNA expression was widespread, detected in ventricular zones/subventricular zone as well as in postmitotic cortical plate neurons (Fig. 7a), and was sharply downregulated at E14.5, E16.5 and E18.5 in *Celsr3*^*−/−*^ mutant compared with control cortical samples (Fig. 7b). At E14.5, Jag1 immunoreactivity was associated with blood vessels in parenchyma and meninges, as described^38^. Using triple IHC with antibodies to Jag1, the neuronal marker Ctip2 and the BP marker Tbr2, Jag1 protein was clearly detected in immature neurons in the intermediate zone and deep cortical plate with little colocalization with BP (Fig. 7c). Importantly, Jag1 immunoreactivity in the intermediate zone and deep cortical plate was specifically downregulated in *Celsr3*^*−/−*^ mutant samples (Fig. 7d). Jag1 immunoreactivity in the intermediate zone and deep cortical plate was also reduced in *Fzd3*^*−/−*^ mutant (Fig. 7f) compared with control tissue (Fig. 7e).

These results suggest strongly that *Celsr3* and *Fzd3* mutant cortical immature neurons and probably some BP express Jag1 less robustly than control cells, resulting in a reduced activation of Notch1 in AP.

**Figure 8:**
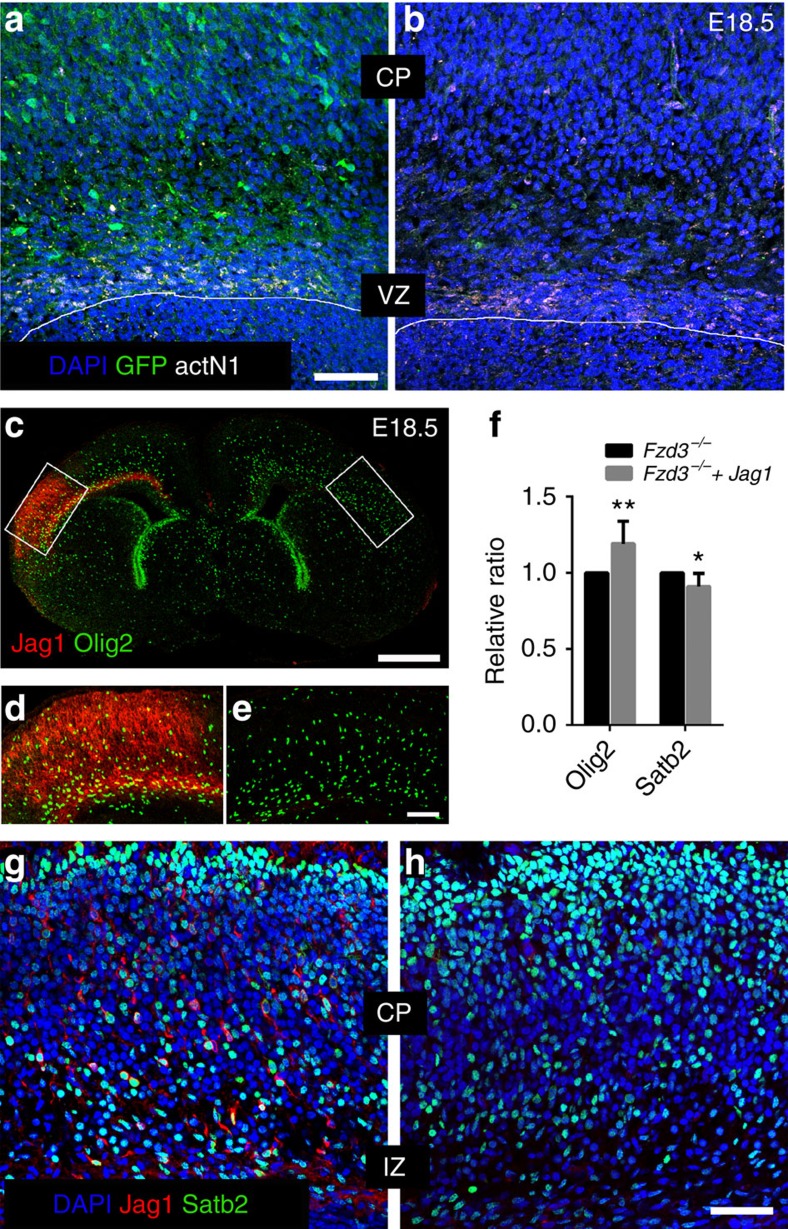
Jag1 overexpression increases Notch activation and Olig2 expression. (**a**,**b**) On electroporation of the Jag1 plasmid and a control GFP plasmid in *Fzd3*^*−/−*^ mutants, GFP expression (green) is observed in CP and migrating cells in intermediate zone (IZ) (**a**). In comparison with the contralateral side (**b**), activation of Notch (actN1, white) is evident in apical progenitors in ventricular zone (VZ) (**a**). Scale bar, 50 μm. (**c**–**h**) Overexpression of Jag1 (red), obtained by electroporation of Jag1 plasmid, results in labelling of more Olig2-positive cells (**c**–**f**) and less Satb2-positive neurons (**f**–**h**) in the electroporated area (**d**) relatively to the contralateral control side (**e**). For quantification in **f** due to variation of electroporation efficiency, cell numbers are normalized to matching contralateral areas. Scale bars, 500 μm (**c**), 100 μm (**d**,**e**), 50 μm (**g**,**h**). Number of samples in **d**: 8 samples of each genotype. Unpaired *t*-test. Error bars: s.d.

If decreased Jag1 expression is implicated in blunted Notch activation and gliogenesis, overexpressing it should rescue the phenotype. To assess this, we electroporated a plasmid encoding Jag1 under the Cdk5r promoter that drives expression in neurons but not AP (ref. 39). Electroporation was carried out at E13.5, and samples were collected at E18.5 and stained with anti-Jag1 to verify overexpression, anti actN1 to check Notch activation, and anti-Olig2 and anti-Satb2 to assess the gliogenesis switch. When Olig2 and Satb2 expression in the electroporated area were compared with that in the contralateral mirror side, a significant increase in the density of Olig2-positive cells (Fig. 8c–f, *P*=0.0024), together with a decreases in the density of Satb2-positive neurons (Fig. 8f–h *P*=0.0197), and the actN1 signal in ventricular zones was clearly increased (Fig. 8a,b).

### Wnt7a upregulates Jag1 in control but not mutant neurons

**Figure 9:**
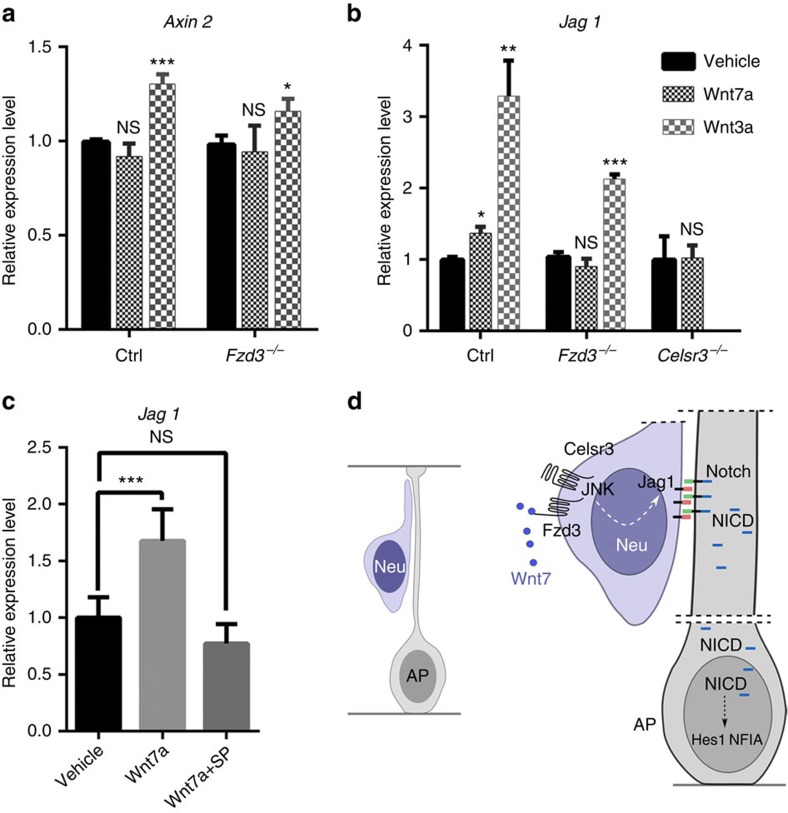
*Fzd3* and *Celsr3* mutant neurons cannot respond to Wnt7a. (**a**) Unlike Wnt3a, Wnt7a does not upregulate *Axin2* mRNA expression when added to cultured cortical control or *Fzd3* mutant neurons. Three cultures for each condition. Unpaired *t*-test. Error bars: s.d. (**b**) Whereas Wnt3a addition upregulates *Jag1* mRNA in both control and *Fzd3* mutant neurons, Wnt7a addition does so in control but not in *Fzd3* or *Celsr3* mutant neurons. Three cultures for each condition. Unpaired *t*-test. Error bars: s.d.(**c**) The upregulation of *Jag1* mRNA induced by Wnt7a is blocked on treatment of cultured neurons with the JNK inhibitor SP600125 (SP). Six cultures for each condition. Unpaired *t*-test. Error bars: s.d. (**d**) Schematic summary of the model. Wnt7 binds to the Celsr3 and Fzd3 complex at the surface of immature neurons (and possibly BP), resulting in JNK-dependent upregulation of Jag1 expression. Jag1 binds to and activates Notch signalling in AP.

We then asked whether Wnt proteins, which bind to Fzd receptors and are known to modify Jag1 expression^40^, could be involved in Jag1 downregulation in *Celsr3* and *Fzd3* mutants. Our RNA-Seq results showed that Wnt7b and Wnt7a are by far the most abundant Wnt factors in the embryonic cortex, followed by Wnt5a, whereas other Wnt factors are expressed at very low, mostly undetectable levels. We tested the putative role of Wnt7 using primary cultures of cortical neurons from E14.5 control, *Fzd3* and *Celsr3* mutant embryos. After 1 day *in vitro*, neurons were placed in serum-free medium for 6 h, exposed to Wnt factors (100 ng ml^−1^) for 5 h and expression of selected mRNA was assessed by qRT-PCR. Wnt7a is known to have mixed, non-canonical and canonical, beta-catenin dependent activity, depending on context. To differentiate between those two activities, we compared the effect of Wnt7a and Wnt3a, a canonical ligand. We measured expression of *Axin2*, a beta-catenin downstream target, and *Jag1*. As predicted, *Axin2* mRNA expression was upregulated on addition of Wnt3a in both control and *Fzd3* mutant neurons, and remained unaffected on addition of Wnt7a, indicating that the latter activated preferentially non-canonical signalling in embryonic cortical neurons (Fig. 9a). Importantly, on Wnt7a treatment, *Jag1* expression was increased in control but not in *Fzd3* or *Celsr3* mutant neurons, whereas addition of Wnt3a induced *Jag1* expression similarly in control and *Fzd3* mutant neurons (Fig. 9b). To confirm that this effect of Wnt7a occurred via non-canonical Wnt signalling with JNK as a downstream effector^41^, we used the c-Jun N-terminal Kinase (JNK) inhibitor SP600125. Addition of SP600125 to cultured neurons counteracted the effect of Wnt7a, and prevented *Jag1* upregulation (Fig. 9c).

Altogether, these data provide strong evidence that Wnt7 facilitates Jag1 expression by cortical neurons, in a Fzd3 and Celsr3 dependent manner.

## Discussion

Our results identify a previously uncharacterized feedback mechanism that regulates the timing of AP fate via Jag1/Notch signalling. Expression of Celsr3 and Fzd3 in immature cortical neurons and possibly BP cells allows them to respond to JNK-dependent, non-canonical Wnt signalling, presumably generated by Wnt7a and Wnt7b. Of note, Wnt7a and Wnt7b are both expressed in the embryonic cortex, with peaks of expression in ventricular zones for Wnt7a, and in intermediate zone and cortical plate for Wnt7b (Eurexpress database: http://www.eurexpress.org). The Wnt7/Celsr3/Fzd3 signal fosters expression of Jag1 in immature neurons, which activates Notch in APs and orchestrates their sequential production of deep and UL cortical neurons, followed by glia (Fig. 9d). Our findings will be briefly discussed in relation to the role of Notch signalling, PCP signalling and Wnt factors in neuro- and gliogenesis.

A role of Notch in AP fate specification is well established^23^,^24^,^25^. It implicates interactions between Delta ligands and Notch receptors in adjacent AP cells^42^, or between Delta on BP and Notch on AP cells^15^. Our data point to the importance of interactions between Jag1 in immature neurons, and Notch in AP, as a feedback mechanism that regulates both neuro- and gliogenesis. As AP cell bodies are located in ventricular zones, at a distance from immature neurons, the Jag1-Notch interaction is postulated to occur between the latter cells and the radial processes of AP. Fine mapping of Jag1 and Notch distribution using super resolution microscopy, or the visualization of local Notch signalling activity in radial processes of AP should advance our understanding of that mechanism.

Like Delta-Notch, PCP signalling is also generated by direct interactions among adjacent neuroepithelial cells and NPC. This ‘epithelial PCP' pathway implicates membrane proteins Celsr1, Fzd3 or Fzd6 and Vangl2, and acts via Dishevelled and Prickle to regulate the organization of the neuroepithelium and AP, with known roles in neural tube closure, neuronal migration and ependymal ciliogenesis^29^,^43^,^44^,^45^. The signal considered in the present work is different. It is mediated by Celsr3 and Fzd3 and involves non-epithelial cells. Celsr3 and Fzd3, probably together with Linx, have key roles in axon guidance^27^,^29^,^46^,^47^,^48^,^49^, but their function in neurogenesis and the gliogenic switch was not previously recognized. The observation that Wnt7 factors trigger the Celsr3/Fzd3 signal in immature neurons argue in favour of a much discussed role of Wnt proteins in PCP signalling. The implication of Wnt in PCP in *Drosophila*, the main PCP model organism, has been heavily debated, although it is gaining increasing recognition^50^. By contrast, studies in vertebrates have long pointed to such a role^51^,^52^. Wnt7a and the PCP effector Vangl2 regulate the orientation and symmetry of mitotic spindles and fate specification of AP, an effect related to epithelial PCP (ref. 53). Our study provides the first indication that Celsr3 and Fzd3 are also required for the response of immature neurons to Wnt factors. Given the similarity of mutant mouse phenotypes, it is reasonable to suggest that Celsr3, Fzd3 and Linx form a large membrane-anchored complex that binds Wnt factors via Fzd, and probably other ligands via Celsr and/or Linx^27^. That complex could be the proximal element of a beta-catenin independent signal in immature neurons to regulate JNK and thereby sustain expression of Jag1. Our results also raise the question of the cellular origin of Wnt7 factors in the telencephalic wall. mRNA expression patterns suggest that Wnt7a is synthetized in the ventricular zones, and Wnt7b in BP and young neurons, indicating that Wnt7 may act in a paracrine manner. Interestingly, in the *Drosophila* eye, Frizzled/PCP signalling specifies photoreceptors R3/R4 and planar polarity, through transcriptional upregulation of Delta in R3 and activation of Notch in R4, possibly in response to an equatorial-polar gradient of Wnt (refs 54, 55, 56, 57), hinting at a fascinating evolutionary conservation of mechanisms.

Unlike Jag-Notch interactions, feedback signals from neurons to AP, which regulate the gliogenic switch, implicate diffusible molecules secreted by neurons and do not require direct neuron-AP interactions^13^. Feedback regulation of UL cortical neurons by DL neurons or other sources has been less extensively studied. Together with the report that Ntf3 can signal in both feedback loops^12^, our work suggests that some mechanisms are indeed common to both, and that specificity may be afforded by the internal status of AP, which is known to vary in a cell autonomous manner^7^,^8^,^9^. The intriguing co-existence of so many mechanisms is in coping with the complexity of regulated sequential cortical neurogenesis and gliogenesis, and requires coordination and interactions with Notch, a master regulator of NPC cell fate. We did not find any indication that the response of NPC to feedback from Fgf, Bmp or cytokines is affected in *Celsr3* or *Fzd3* mutants. Regulatory mechanisms are hence likely to operate in parallel and it will be challenging to understand how they are integrated *in vivo* during development, and to harness their potential for regeneration.

## Methods

### Animals

Mice were bred in standard conditions and animal procedures were carried out in accordance with European guidelines and approved by the animal ethics committee of the Université catholique de Louvain. *Celsr3* and *Fzd3* mutant mice are on mixed C57Bl/6 and CD1 background, and control mice are heterozygous or wild-type littermates.

### IHC, antibodies and *in situ* hybridization

Following intracardiac perfusion with 4% paraformaldehyde (PFA) in PBS, brains were removed and fixed in PFA for 4 h or overnight at 4 °C. They were cryopreserved in 30% sucrose and embedded in optimal cutting temperature compound. Sections (10 μm) were prepared using a cryostat. Antigen retrieval was performed by incubating sections in 0.01 M sodium citrate (pH 6.0) for 20 min at 90–95 °C. Blocking was in 5% normal goat or horse serum in PBS with 0.3% Triton X-100. Primary antibodies were diluted in PBS containing 0.3% Triton X-100, 5% normal goat or horse serum, with overnight incubation. Incubation with secondary antibodies was for 1–2 h at room temperature. Primary antibodies were: rabbit anti-Tbr1 (1:200, Abcam ab31940), rabbit anti-Tbr2 (1:200, Abcam ab23345), rat anti-Ctip2 (1:500, Abcam ab18465), mouse anti-Satb2 (1:500, Abcam ab51502), rabbit anti-Olig2 (1:1,000, Millipore AB9610), mouse anti-GFAP (1:200, Sigma G3893), rabbit anti-cleaved Notch1 Val1744 (1:100, Cell signaling 4147), goat anti-Jag1 (1:100, Santa Cruz sc6011), rabbit anti-Dll1 (1:100, Santa Cruz sc9102), rabbit anti-Caspase3 (1:200, Cell signaling 9661), mouse anti-Ki67 (1:1,000, BD Biosciences 556003), rat anti-BrdU (1:200, Serotec MCA2060GA), rabbit anti-Cux1 (1:200, Santa Cruz sc13024), rabbit anti-Pax6 (1:200, Covance PRB-278P), mouse anti-βIII tubulin/TuJ1 (1:1,000, Covance MMS-435P-0250). Secondary antibodies conjugated to Alexa 488, 568 or 647 were goat anti-mouse (Invitrogen A21141, A21144 and A21050), donkey anti-mouse (Invitrogen A21202, A10037 and A31571), goat anti-rabbit (Invitrogen A11034, A11036 and A21070), donkey anti-rabbit (Invitrogen A21206, A10042 and A31573), and goat anti-rat labelled with Alexa 568 (1:500, Invitrogen A11077), all used at 1:500 dilution. Nuclei were stained with 4,6-diamidino-2-phenylindole. Images were acquired with an Olympus FV1000 confocal microscope and edited with Fluoview (Olympus) and Photoshop (Adobe Systems).

For *in situ* hybridization, the *Jag1* probe was cloned using primers: Jag1 F: 5′-ATGCCTCGAGAATTGAGGAATTTGAATATC-3′ and Jag1 R: 5′-CGGGGTACCCCATCCGGTTCAAGCTCTG-3′. The digoxigenin-labelled probe was produced from the linearized plasmid with T7 RNA polymerase, using a DIG RNA labeling kit (SP6/T7; Sigma 11175025910) according to the manufacturer instructions. Cryostat sections were treated with 1 μg ml^−1^ proteinase K in 0.1 M Tris-HCl, pH 8 and 10 mM EDTA, rinsed in diethylpyrocarbonate treated water and acetylated for 10 min at room temperature in 0.25 M acetic anhydride-0.1 M triethanolamine. Slides were incubated overnight at 65 °C in a humid chamber with denatured probes (1 μg ml^−1^) in hybridization solution (50% formamide, 10% dextran sulphate, 0.3 M NaCl, 20 mM Tris-HCl, pH 7.5, 5 mM EDTA, 1 × Denhardt's solution, 0.6 mg ml^−1^ yeast tRNA, and 0.1% SDS). Slides were washed for 30 min at 65 °C in 50% formamide- 2 × SSC, rinsed in 2 × SSC and treated for 1 h at 37 °C with 1 μg ml^−1^ RNAseA in NaCl-Tris-EDTA buffer (0.5 M NaCl, 10 mM Tris-HCl, pH 7.5, 5 mM EDTA). Slides were washed in 2 × SSC and 0.2 × SSC at 65 °C for 1 h each, blocked with 20% sheep serum and incubated overnight with alkaline phosphatase-coupled digoxigenin antibodies (1/2,000; Roche 11333089001).

### Validation of Nex-Cre-induced gene inactivation

Nex-Cre is predicted to express Cre in cortical neurons but not AP. To check Cre activity, 200-μM-thick vibratome sections were prepared to cut the cortex tangentially into deep and upper regions enriched in AP and neurons, respectively. RT-PCR was carried out using primers 5′-TTCCACCCTATGGTGAACCT-3′ and 5′-CTACTGCACTCCATATCTTCAGG-3′ in *Fzd3* exon 3 that should be deleted on Cre expression.

### qRT-PCR and RNA-Seq

Total RNA was extracted from brain cortex or cultured neurons using an RNeasy mini kit (Qiagen). Reverse transcription was performed with a Promega reverse transcription system (A3500). qPCR was carried out using IQ SYBR green supermix system (Biorad). Primer sequences are provided in Supplementary Table 1. For RNA-Seq, E16.5 control, *Fzd3* and *Celsr3* mutant cortical tissue (three of each genotype) was dissected carefully. Total RNA was extracted, and sequencing and bioinformatics analysis were carried out by Beckman Coulter Genomics. Results have been deposited at NCBI/GEO under reference GSE77081.

### Western blot

Cortices were homogenized in lysis buffer containing 50 mM Tris-HCl (pH 7.5), 150 mM NaCl, 10 mM EDTA, 1% Triton × 100 and protease inhibitors (Complete, Roche). Cell lysates were centrifuged at 13,000*g* for 15 min at 4 °C. Supernatant (40 μl) was mixed with 10 μl 5 × SDS-loading buffer and heated at 95 °Cfor 5 min. Proteins were separated by SDS–polyacrylamide gel electrophoresis and transferred to nitrocellulose membranes (GE healthcare). Membranes were blocked with 5% fat-free dry milk or 5% BSA and incubated overnight at 4 °C with the following antibodies: rabbit anti-Notch1(V1744; 1:1,000, Cell Signaling 4147); rabbit anti-phospho-Erk1&2 (1:2,000; Cell Signaling 4370); mouse anti-GFAP(1:1,000; Sigma G3893); chick anti- Gapdh (1:5,000; Millipore AB2302); rabbit anti-phospho-Smads (1:2,000; Santa Cruz sc12353); rabbit anti-phospho-Stat3 (1:1,000; Cell signaling 9145). Full-size western blots used in Figs 4i and 6c and Supplementary Fig. 8 are shown in Supplementary Fig. 10.

### Cell cycle study and lineage tracing with BrdU

BrdU or/and EdU (50 μg g^−1^ body weight) were injected intraperitoneally to pregnant dams. For estimation of cell cycle exit, IHC for BrdU and Ki67 was used to calculate ratio of BrdU^+^ Ki67^−^ to total BrdU^+^ cells. For estimation of S-phase and cycle length^37^ EdU labelling was carried out using a Click-iT EdU Alexa 488 Imaging kit (Life Technology C10337) according to the manufacturer's instructions. For lineage tracing, antibodies against BrdU, Olig2 and Satb2 were used.

### *In utero* electroporation

The Jag1 coding sequence was recovered from plasmid Addgene 17336 (ref. 58) encoding the rat Jag1 (mouse and rat Jag1 proteins are 99% identical), and cloned in the Cdk5r vector (kindly provided by Dr Paola Arlotta). The construct was verified by sequencing. The plasmid was electroporated together with a green fluorescent protein (GFP) encoding plasmid. *In utero* microinjection and electroporation was performed at E13.5, using pregnant *Fzd3*^*+/−*^ females; mutant embryos were identified by their looptail phenotype. Foetuses were examined at E18.5 and genotypes were confirmed using tail clips. Needles for microinjection were pulled from Wiretrol II glass capillaries (Drummond Scientific) and calibrated for 1 μl injections. DNA solutions were mixed in 10 mM Tris, pH 8.0, with 0.01% Fast Green. Forceps-type electrodes (Nepagene) with 5 mm pads were used for electroporation (five 50-ms pulses of 30 V)^59^.

### Primary neuronal culture and Wnt ligands treatment

Cortices from E14.5 embryos were dissociated mechanically by gentle pipetting. In all, 10^6^ cells were plated on a coverslip coated with poly-D-lysine (BD), in neurobasal medium with Glutamax (Invitrogen), B27 (Invitrogen), N2 (Invitrogen) and 5% foetal bovine serum. After 1 day *in vitro*, neurons were serum-deprived for 6 h and then incubated with Wnt3a or Wnt7a (100 ng ml^−1^, R&D) for 5 h. Total RNA was extracted and gene expression levels estimated by qRT-PCR. For JNK inhibition, SP600125 (50 μM, Sigma) was added together with Wnt7a.

### Statistics

All animal experiments concerned at least three animals, and all other experiments were carried out at least in triplicate, as indicated in legends. Error bars are s.d. Unpaired Student's two tailed *t*-Test were used, with significance levels * for *P*<0.05, ** for *P*<0.01 and *** for *P*<0.001.

## Additional information

**Accession code:** RNA-seq results have been deposited at NCBI/GEO under reference GSE77081.

**How to cite this article:** Wang, W. *et al*. Feedback regulation of apical progenitor fate by immature neurons through Wnt7–Celsr3–Fzd3 signalling. *Nat. Commun.* 7:10936 doi: 10.1038/ncomms10936 (2016).

## Supplementary Material

Supplementary Information Supplementary Figures 1-10 and Supplementary Table 1
