# Supplementary material for: Feedback regulation of apical progenitor fate by immature neurons through Wnt7–Celsr3–Fzd3 signalling
Source: Nat Commun. 2016 Mar 4;7:10936. doi: 10.1038/ncomms10936 (PMC4786577; doi:10.1038/ncomms10936)
Supplement: Supplementary Information — Supplementary Figures 1-10 and Supplementary Table 1 [file ncomms10936-s1.pdf]

Supplementary Figures and Table

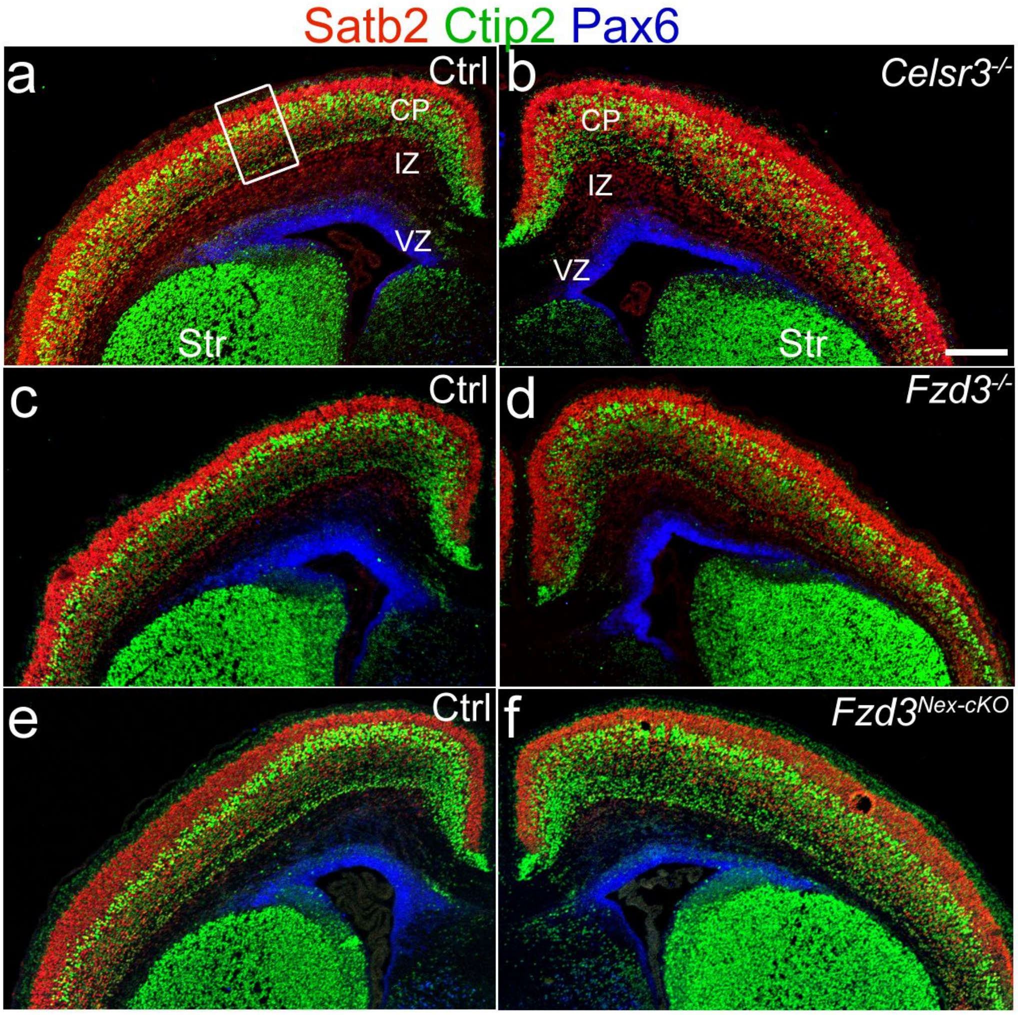

Supplementary Figure 1

Cortical neurons are increased in mutants.

(**a to f**) Low power view of coronal sections in E18.5 control (**a, c, e**) and *Celsr3*$^{-/-}$ (**b**), *Fzd3*$^{-/-}$ (**d**) and conditional *Fzd3*$^{Nex\text{-}cKO}$ (**f**) mutant telencephalon, stained for Satb2 (red), Ctip2 (green) and Pax6 (blue). CP: cortical plate; VZ: ventricular zone; Str: striatum. The frame in **(a)** shows the regions selected for illustrations shown in **Figure 1 (a,b,d,e,g,h)**, **Figure 2 (a,b,d,e,g,h,j,k), Figure 8 (e,f)** and **Supplementary Figure 3(a,b,c)**. **Scale** bar = 250 µm

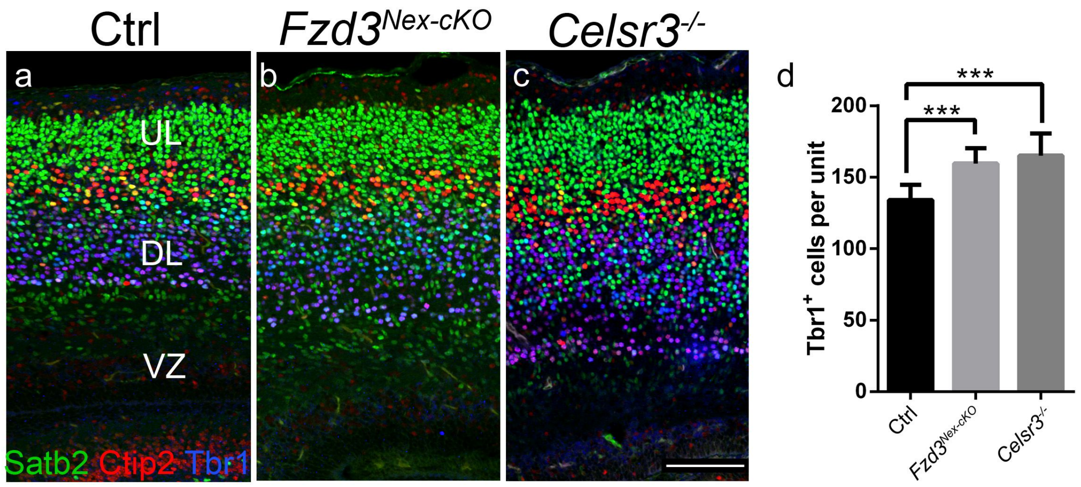

Supplementary Figure 2

Deep layer neurons (Tbr1^{+}) are increased in mutant cortex.

(**a–d**) Deep layer (DL) marker Tbr1 is increased in *Celsr3*$^{-/-}$ and conditional *Fzd3*$^{Nex\text{-}cKO}$ mutant cortical plate (CP) at E18.5. VZ: ventricular zone. Scale bar = 100 μm. Number of samples for (d) 13 Ctrl, 7 *Fzd3*$^{Nex\text{-}cKO}$ and 9 *Celsr3* sections from 3 animals of each genotype. Unpaired t-test. Error bars : SD.

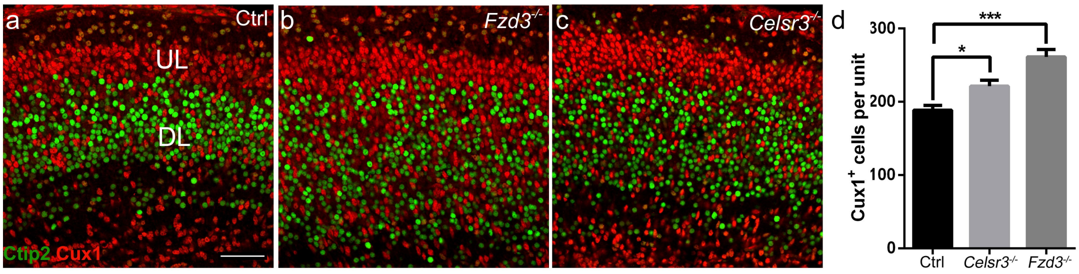

Supplementary Figure 3

Upper layer neurons (Cux1$^+$) are increased in mutant cortex.

(**a–d**) IHC for Cux1, an upper layer (UL) marker like Satb2, shows that, like DL, UL neurons are increased in number in *Celsr3*$^{-/-}$ and *Fzd3*$^{-/-}$ mutant cortex. Scale bar = 50 µm. Number of samples for (d) 8 Ctrl, 4 *Celsr3* and 8 *Fzd3* sections from 3 animals of each genotype. Unpaired t-test. Error bars : SD.

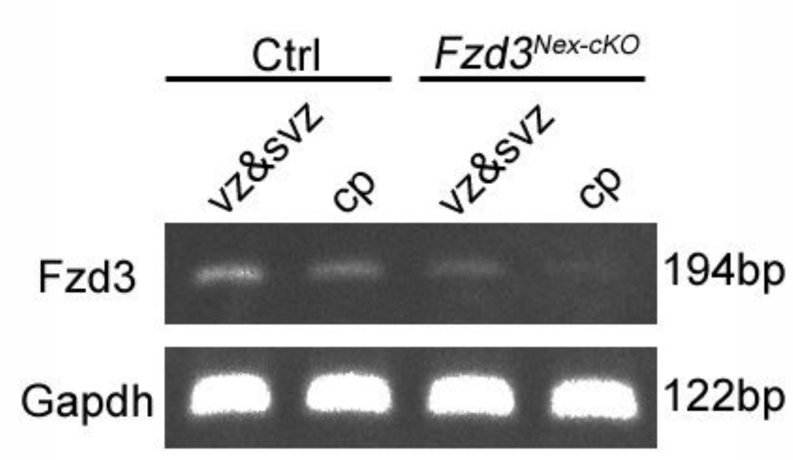

Supplementary Figure 4

Validation of Nex-Cre induced *Fzd3* inactivation

Samples enriched in NPC (vz & svz) and in cortical neurons (cp) were dissected from Ctrl and *Fzd3*$^{Nex\text{-}cKO}$ animals at E17.5. RT-PCR was carried out with primers in *Fzd3* exon 3, which is deleted upon Cre expression and with *Gapdh* primers as control.

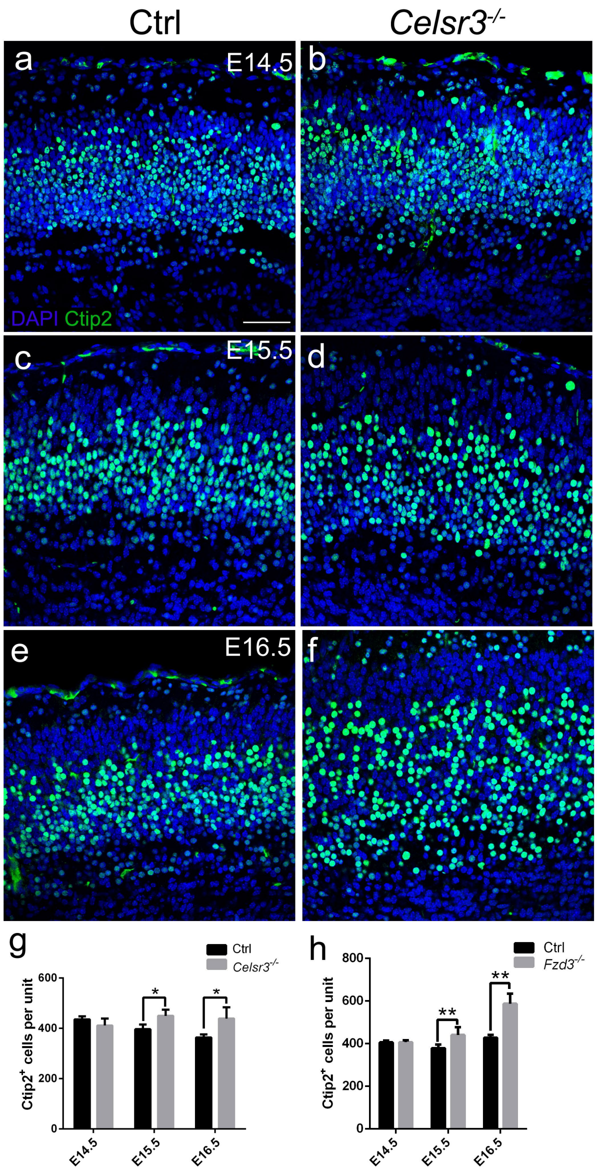

Supplementary Figure 5

Time-course analysis shows the generation of DL neurons is prolonged.

(**a–f**) IHC with the DL marker Ctip2 shows that the *Celsr3*$^{-/-}$ mutant cortical plate is similar to the control one at E14.5, and that the increase in DL neurons in the mutant are initially seen at E15.5 and become more evident at E16.5. Scale bar = 50 µm. (**g, h**) Quantification in *Celsr3*$^{-/-}$ (**g**) and *Fzd3*$^{-/-}$ (**h**) mutants. 3 embryos of each genotype and stage. Unpaired t-test. Error bars : SD.

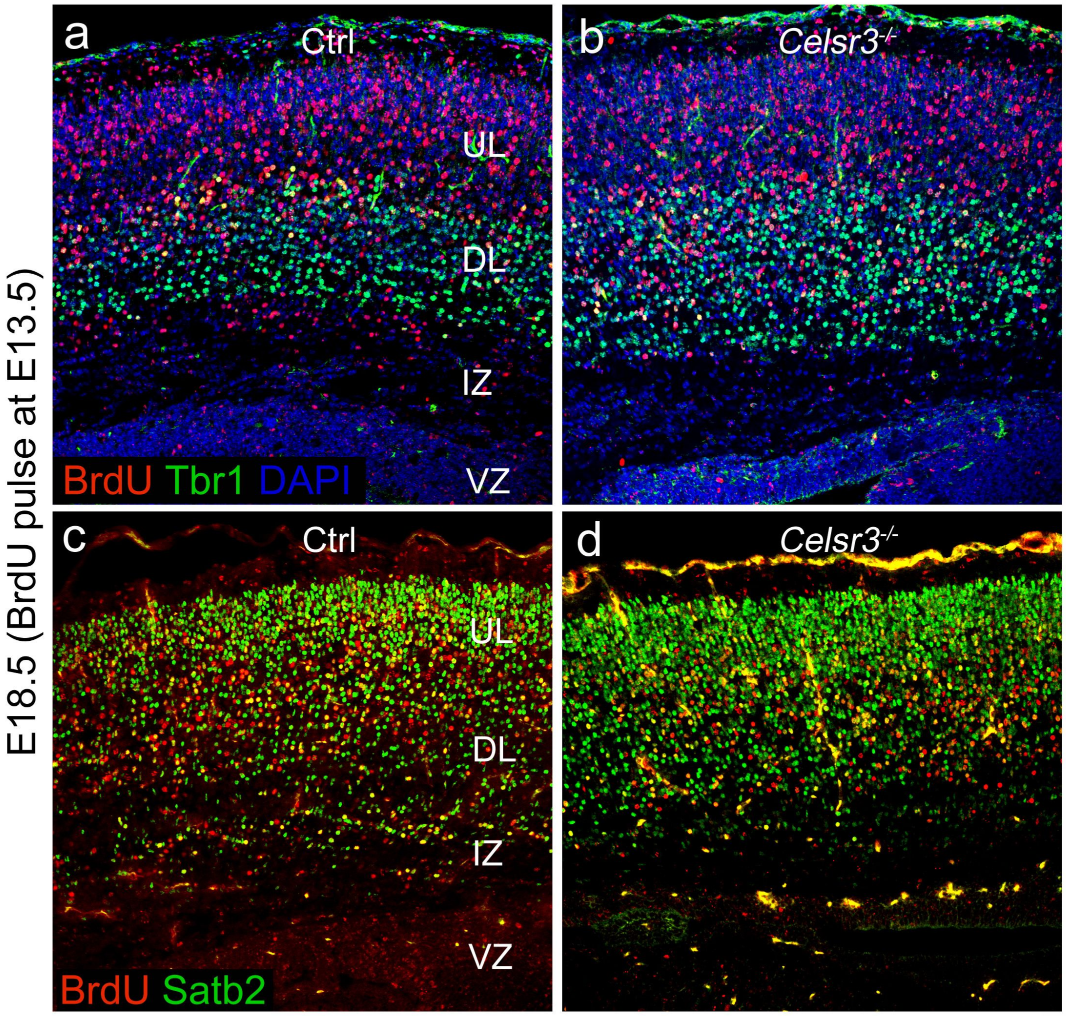

Supplementary Fig. 6

Generation of deep layer neurons

a–d: Low magnification views of the dorsal telencephalic wall at E18.5 in control (Ctrl) and *Celsr3*$^{-/-}$ mutants, with expression of BrdU (red), Tbr1(green) and DAPI as nuclear stain in (a,b). VZ: ventricular zone; IZ: intermediate zone; DL: deep cortical layer; UL: upper cortical layer.

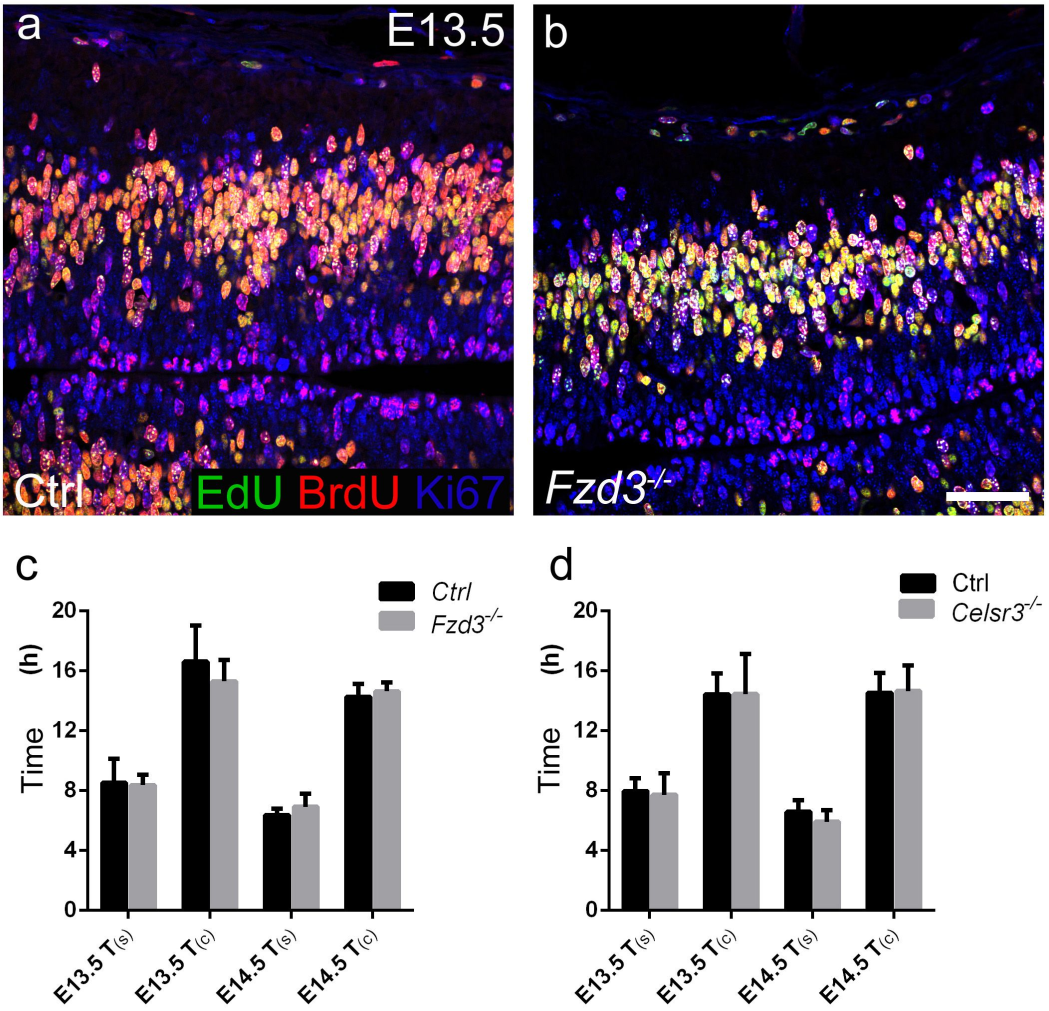

Supplementary Figure 7

Comparison of NPC cell cycle length

(a, b) Example of triple staining for EdU (green), BrdU (red) and Ki67 (blue) shows no difference between Ctrl and mutants at E13.5. Scale bar = 50 μm.

(c, d) Cell cycle length T(c) and S-phase length T(s), estimated as described in Methods are not significantly different in Ctrl, *Fzd3*$^{-/-}$ and *Celsr3*$^{-/-}$ mutant cortical tissue at E13.5 and E14.5. Number of samples: 6 sections from 3 embryos at each stage. Unpaired t-test. Error bars : SD.

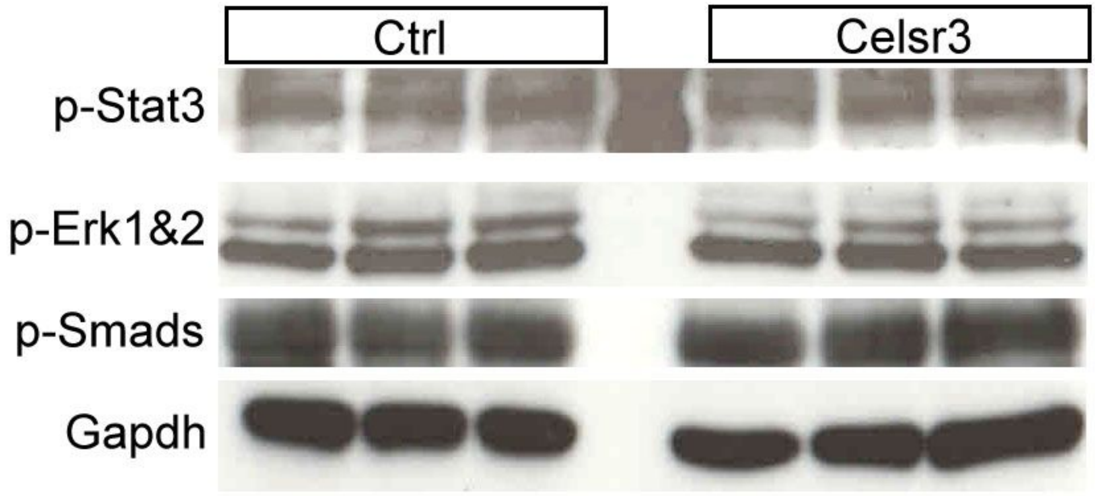

Supplementary Figure 8

Western blot analysis of key signal pathways involved in gliogenic switch.

Western blot analysis of control and *Celsr3*$^{-/-}$ mutant cortex at E18.5 with pStat3 (Jak/Stat signaling), pErk1&2, pSmads (BMP signaling) and Gapdh as control, shows no differences between genotypes.

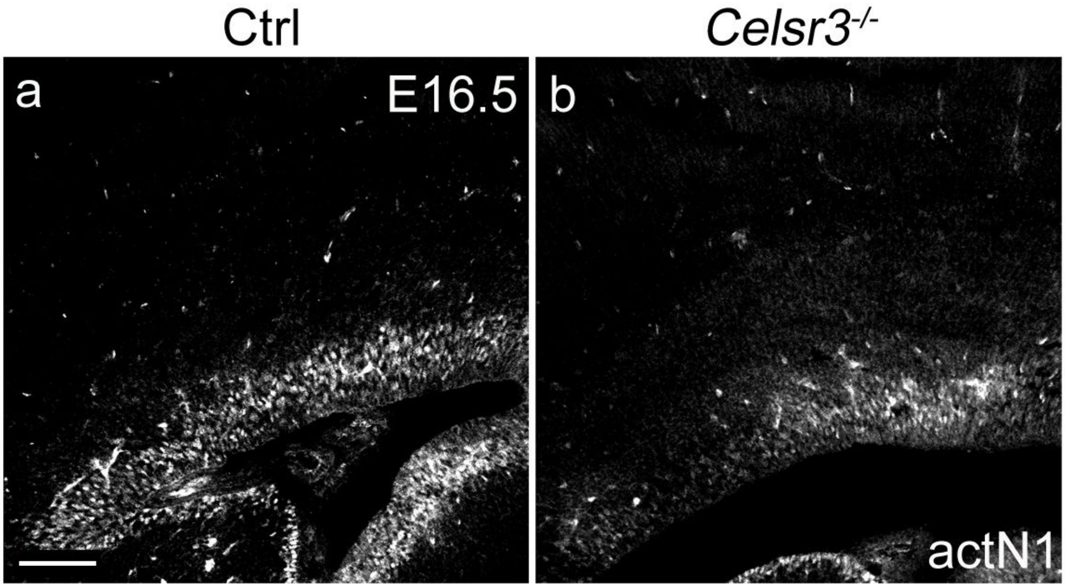

Supplementary Figure 9

Notch activity

The signal of active Notch (actN1) is decreased in *Celsr3*$^{-/-}$ mutant tissue (b) compared to control (Ctrl, a) at E16.5. Scale bar = 50 µm.

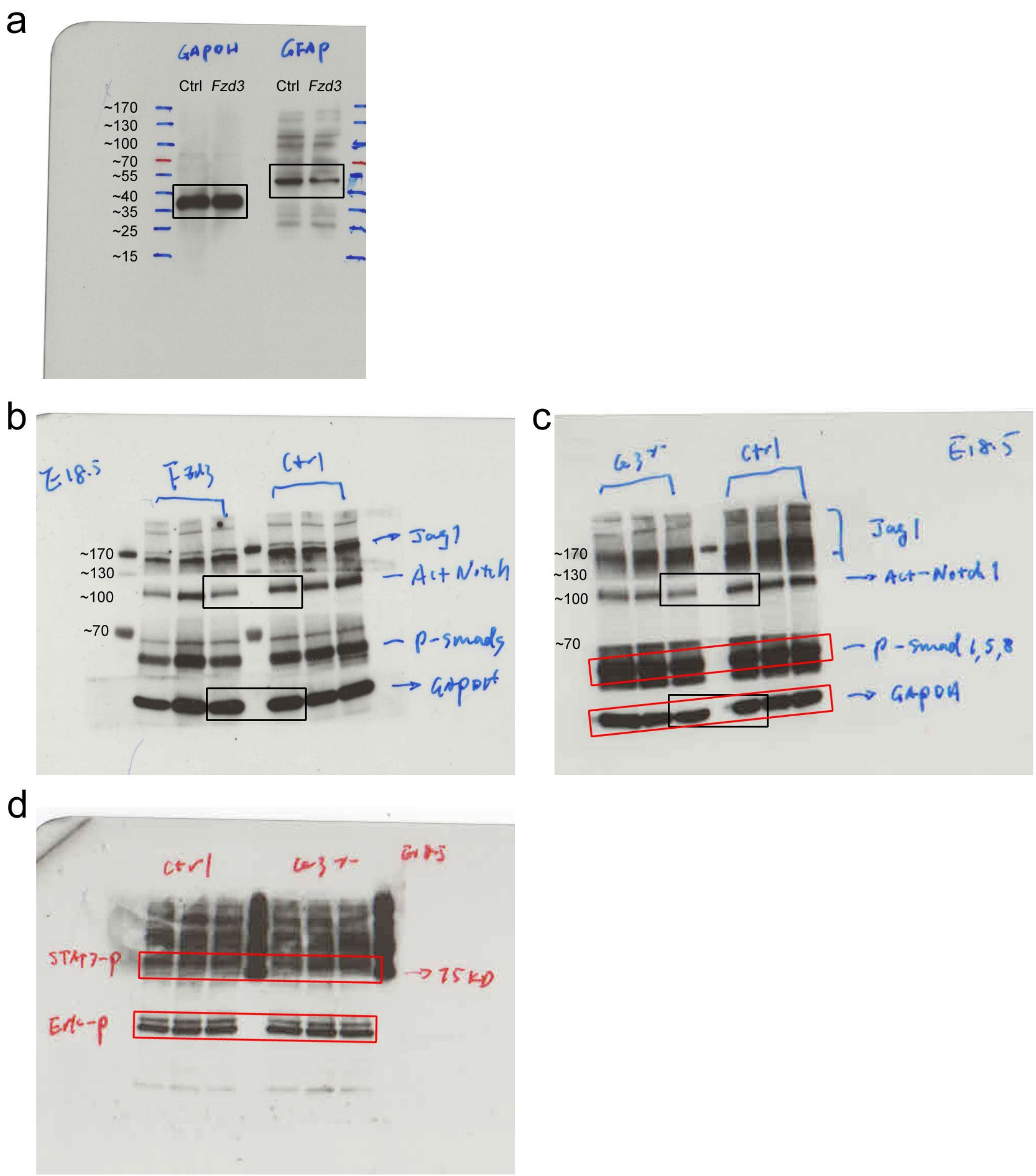

Supplementary Fig. 10

Original western blots

Full size western blots used to generate Fig 4i (a), Fig. 6c (b,c, black frames) and Supplementary Fig. 8 (c,d, red frames).

Dll1 F	AAGGATATAGCCCCGATGAATGC
Dll1 R	TGCTAACTCTGAGAGAACCAGCTTCG
Jag1 F	CAGTGCCTCTGTGAGACCAA
Jag1 R	AGGGGTCAGAGAGACAAGCA
Jag2 F	ATCAGCAGCTCCTCATCTGG
Jag2 R	CGTCGTCATTCCCTTTCAGT
Hes1 F	CCAGCCAGTGTCAACACGA
Hes1 R	AATGCCGGGAGCTATCTTTCT
Hes5 F	AAGAGCCTGCACCAGGACTA
Hes5 R	CGCTGGAAGTGGTAAAGCA
NFIA F	CTTTGTACATGCAGCAGGAC
NFIA R	TTCCTGCAGCTATTGGTGTTT
Notch1 F	GATGGCCTCAATGGGTACAAG
Notch1 R	TCGTTGTTGTTGATGTCACAGT
GAPDH F	AGGTCGGTGTGAACGGATTTG
GAPDH R	TGTAGACCATGTAGTTGAGGTCA
Axin2 F	TGACTCTCCTTCCAGATCCCA
Axin2 R	TGCCCACACTAGGCTGACA

Supplementary Table 1. Primers for qPCR
